# Bone fracture among people living with HIV: A systematic review and meta-regression of prevalence, incidence, and risk factors

**DOI:** 10.1371/journal.pone.0233501

**Published:** 2020-06-04

**Authors:** Iqbal Pramukti, Linlin Lindayani, Yen-Chin Chen, Chun-Yin Yeh, Ta-Wei Tai, Susan Fetzer, Nai-Ying Ko

**Affiliations:** 1 Department of Nursing, International Doctoral Program in Nursing, College of Medicine, National Cheng Kung University, Tainan, Taiwan; 2 Faculty of Nursing, Universitas Padjadjaran, West Java, Indonesia; 3 Sekolah Tinggi Ilmu Keperawatan PPNI Jawa Barat, Bandung, Indonesia; 4 Department of Nursing, National Cheng Kung University Hospital, College of Medicine, National Cheng Kung University, Tainan, Taiwan; 5 Department of Nursing, College of Medicine, National Cheng Kung University, Tainan, Taiwan; 6 Department of Computer Science and Information Engineering, National Cheng Kung University, Tainan, Taiwan; 7 Departments of Orthopedics, National Cheng Kung University Hospital, College of Medicine, National Cheng Kung University, Tainan, Taiwan; 8 Skeleton Materials and Bio-compatibility Core Lab, Research Center of Clinical Medicine, National Cheng Kung University Hospital, College of Medicine, National Cheng Kung University, Tainan, Taiwan; 9 Southern New Hampshire Medical Center, Nashua, New Hampshire, United States of America; Medical College of Wisconsin, UNITED STATES

## Abstract

**Introduction:**

People living with HIV (PLWH) had a higher prevalence and incidence rate of bone fracture compared to general population. Although several studies have explored this phenomenon, the prevalence and incidence rate of fracture were varied.

**Objective:**

The aim of the study is to determine and analyze the pooled prevalence, incidence rate of fracture and fracture risk factors among people living with HIV (PLWH).

**Methods:**

PubMed, Cochrane Library, CINAHL with full Text, and Medline databases for studies published up to August 2019 were searched. Studies reporting the prevalence or incidence of fracture among PLWH were included. Study quality was assessed using the Joanna Briggs Institute (JBI) appraisal tool. A meta-analysis with random-effects model was performed to determine pooled estimates of prevalence and incidence rates of fracture. A meta-regression was performed to determine the source of heterogeneity.

**Results:**

The pooled estimated prevalence of fracture among PLWH was 6.6% (95% CI: 3.8–11.1) with pooled odds ratio of 1.9 (95%CI: 1.1–3.2) compared to the general population. The pooled estimates of fracture incidence were 11.3 per 1000 person-years (95% CI: 7.9–14.5) with incidence rate ratio (IRR) of 1.5 (95% CI: 1.3–1.8) compared to the general population. Risk factors for fracture incidence were older age (aHR 1.4, 95% CI: 1.3–1.6), smoking (aHR 1.3, 95% CI: 1.1–1.5), HIV/HCV co-infection (aHR 1.6, 95% CI: 1.3–1.9), and osteoporosis (aHR 3.3, 95% CI: 2.2–5.1).

**Conclusions:**

Our finding highlights a higher risk of fracture among PLWH compared to the general population. Osteoporosis, smoking and HIV/HCV coinfection as the significant modifiable risk factors should be prioritized by the HIV health providers when care for PLWH.

## Introduction

The success of highly active antiretroviral therapy (HAART) over the past decades has increased the life expectancy among people living with HIV (PLWH) [1]. PLWH have increased co-morbidities, including bone fractures, which have emerged to become an important healthcare issue [2]. PLWH experience a greater risk of fractures, ranging from 1.6 to 3 times that of the general population [3]. The risk of fracture among PLWH has been associated with an increasing rate of osteoporosis due to accelerated bone loss [4, 5], bone microstructure alterations, and a reduction in bone mineral density [6]. The International Osteoporosis Foundation noted that bone fractures result in chronic pain, long-term disability, and even death [7].

A proactive approach in the prevention and treatment program is needed to minimize the consequence of bone loss and morbidity associated with fractures among PLWH [8]. Therefore, identifying the prevalence, incidence, and risk factors for fractures among PLWH is important to evaluate a prevention program. However, the current prevalence and incidence rate data are varied. Previous studies have focused on the high prevalence of osteoporosis among PLWH (15%) but not fracture risk [9]. A cohort study of PLWH identified a fracture incidence rate of 31 per 1000 person-year [10], however, a rate of 13.5 per 1000 person-year was reported in another study [3]. Arnsten’s study [10] only included older subjects (≥ 49 years), while older age was found as a risk factor for fractures among PLWH in a study by Dong et al [11].

The risk factors for fracture among PLWH remain unclear. Previous studies have been limited to select HIV populations or have included osteoporosis, not fractures, as an outcome. Earlier studies on fracture risk factors among PLWH showed a varied result. A study by Bedimo et al. found the exposure to tenofovir (TDF) regimen was the risk factors for fracture [12]. Meanwhile, Arnsten et al. found low bone mineral density (BMD) as a significant risk factor for fracture [10]

The variability of conclusive information on fracture among PLWH requires additional study. The study aimed to conduct a systematic review, and meta-regression to determine the pooled prevalence, incidence rate of fracture and fracture risk factors among PLWH.

## Methods

### Search strategy

Using PRISMA guidelines for a systematic review, databases were searched up to 9 August 2019 included PubMed, Cochrane Library, CINAHL with full text, and MEDLINE [13]. The terms used in the searches varied according to the database utilized, thus included *HIV*, *Human Immunodeficiency Virus*, *Acquired Immunodeficiency Syndrome*, *bone fracture*, *bone fragility*, *broken bone*, *osteoporosis*, *bone density*, *prevalence*, *incidence*, *risk factor*, *and epidemiology*.

A study was eligible for inclusion if it included HIV-infected subjects aged 16 years old or above and reported fracture prevalence or incidence rate data. Cross-sectional, cohort, and case-control studies with or without a control group were included. Studies were excluded if they were not in English. Two researchers (IP, LL) independently screened all titles, abstracts, and full texts and appraised study quality. The disagreement was resolved by a third researcher (CY).

### Data extraction

Data extraction included author, year of publication, study location, study design, demographics, sample size, prevalence and incidence rates of fracture, and method of fracture classification. Prevalence of fracture was expressed as a percentage of fracture among HIV positive patients from the total of HIV positive patients. Incidence was expressed as the number of fractures divided by the period of risk for all included subjects. Prevalence and incidence rate data were obtained from the studies selected. All reported cases of osteoporosis in the included studies were diagnosed by using Dual-Energy X-ray Absorptiometry (DXA). When data was not provided, the prevalence rate was calculated by determining the number of fractures of the total HIV positive sample.

### Quality assessment

Study quality was assessed using the Joanna Briggs Institute (JBI) critical appraisal checklist tool for a prevalence study [14] The checklist consists of nine questions with four categories of answers: yes, no, unclear, and not applicable (insufficient data). “Yes” is scored as 1 and “no” is scored as 0, with a total quality score ranging from 0–9.

### Data analysis

Pooled estimates used a random-effects model because of heterogeneity indicated by the I^2^ statistic (values of <25%, 25–75%, and >75% representing low, medium, and high heterogeneity, respectively) [15]. Subgroup analysis and meta-regression were performed to determine potential sources of heterogeneity. Publication bias test were performed using Egger test and Funnel plot. Statistical analysis was conducted using the Comprehensive Meta-Analysis Software (Version 3.0 Biostat, Englewood, New Jersey, USA).

## Results

**Fig 1:**
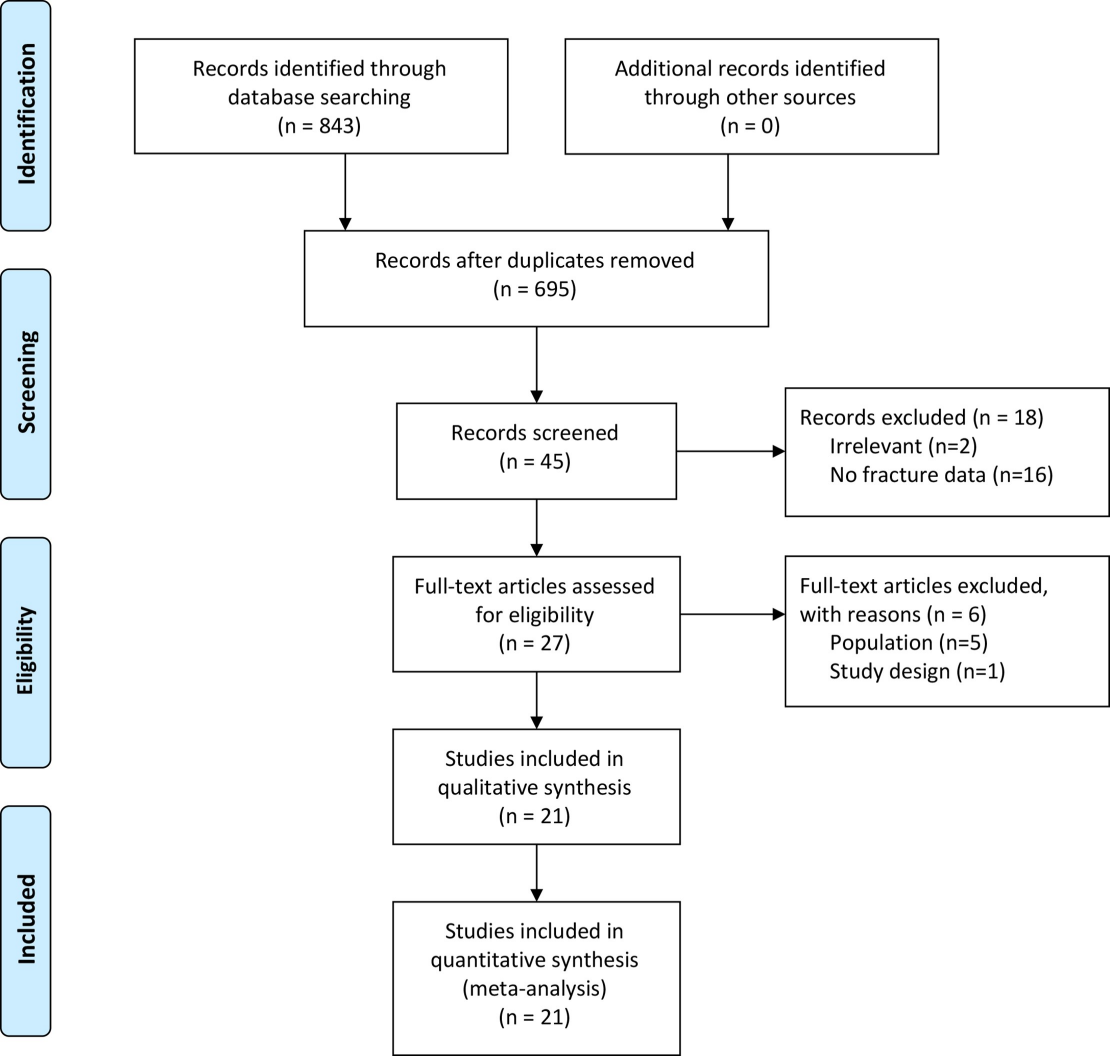
Flow diagram describing article selection according to PRISMA guidelines.

Four databases provided 843 articles from the year 2000 to February 2017 (Fig 1). After excluding duplicates, and applying inclusion and exclusion criteria, 45 articles remained. After full-text examination, 21 articles remained for quality appraisal with all receiving a quality score above 7. Twelve studies received a score of 8 out of 9, and the remaining received a score of 9 out of 9 (Supporting information). Overall, the study quality was acceptable.

### Study characteristics

Published between 2008 and 2017, all 21 studies, were conducted in developed countries. Eleven were in Europe, nine were in the United States, and the other one was a multi-countries study conducting in the European country, Israel, and Argentina (Table 1). Thirteen studies reported the prevalence and incidence data [16–28] while 8 studies reported prevalence data only [6, 29–35].

**Table 1:** Characteristic of studies reporting prevalence or incidence of bone fracture among PLWH (n = 21).

Author (year)	Country	Study design	Population	Sample size (N)	Male (%)	Race (%)	Age (years)	HAART exposure (%)	Fracture ascertainment	Fracture prevalence (%)	Fracture incidence (per 1000 py)
Ciulini et al (2017) [6]	Italy	Cross-sectional	All HIV (+)	141	87.2	86.5 Caucasian	43.0, 37.0–52.0	93.6	Semiquantitative/ morphometric	13.5	-
						13.5 Other	(median, IQR)				
Borges et al (2017) [16]	Multi-countries	Prospective cohort	All HIV (+)	11820	75.0	86 White	49.0	96.4	Self-report	4.2	7.2
						3 Asia					
						5 Black					
						6 Other					
Battalora et al (2016) [17]	USA	Prospective cohort	All HIV(+)	1006	83.2	67 White	43.0, 36.0–49.0	96.0	Self-report	8.4	20.9
						21.1 Black	(median, IQR)				
						8.9 Hispanic					
						3 Other					
Sharma et al (2015) [18]	USA	Prospective cohort	HIV(+) women	1713	0	24 White	40.0, 34.0–46.0	63.0	Self-report	17.5	21.9
						59 Black	(median, IQR)				
						17 Hispanic					
Gazzola et al (2015) [29]	Italy	Cross-sectional	All HIV (+)	194	73.0	91.0 Caucasian	49.0, 40.0–51.0	71.0	Semiquantitative/ morphometric	12.4	
							(median, IQR)				
Byrne et al (2015) [19]	USA	Retrospective cohort	All HIV (+)	96253	61.7	27 White	40.9	100	ICD codes	0.8	2.1
						56.1 Black					
						21.8 Hispanic					
						5.1 Other					
Short et al (2014) [30]	UK	Cross-sectional	HIV (+) men	168	100	97 Caucasian	45.0, 38.0–51.0	78.0	Self-report	13.7	-
							(median, IQR)				
Porcelli et al (2014) [31]	Italy	Cross-sectional	All HIV (+)	131	71.0	-	51.0, 36.0–75.0	-	Semiquantitative/ morphometric	26.7	-
							(median, IQR)				
Borderi et al (2014) [32]	Italy	Cross-sectional	All HIV (+)	202	68.0	13.8 Caucasian	51.0	86	Semiquantitative/ morphometric	23.3	-
Peters et al (2013) [33]	UK	Cross-sectional	All HIV (+)	222	60.0	48.0 Caucasian	45.6, 9.3	85	Self-report	20.3	-
						4 Asian	(mean, SD)				
						38.0 Africa					
						10 Other					
Maalouf et al (2013) [20]	USA	Retrospective cohort	All HIV (+)	56660	98.2	34.8 White	43.5, 36.7–51.8	64.2	ICD codes	1.4	2.07
							(median, IQR)				
Guerri et al (2013) [21]	Spain	Retrospective cohort	All HIV (+)	2489	75.3	-		-	ICD codes	2.0	8.03
Yin et al (2012) [22]	USA	Prospective cohort	All HIV (+)	4640	83.4	48.0 White	39.0, 33.0–45.0	99.7	Self-report	2.3	4.0
						28.7 Black	(median, IQR)				
						20.4 Hispanic					
						1.8 Asian					
						1.2 Other					
Torti et al (2012) [34]	Italy	Cross-sectional	HIV (+) men	160	100	-	53.0, 42.0–71.0	78.1	Semiquantitative/ morphometric	26.9	-
							(median, IQR)				
Lo re et al (2012) [23]	USA	Retrospective cohort	All HIV (+)	95827	63.1	27.3 White	39.0, 33.0–46.0 (median, IQR)	-	ICD codes	0.8	1.95
						44.4 Black					
						8.5 Hispanic					
						19.9 Other					
Hansen et al (2012) [24]	Denmark	Prospective cohort	All HIV (+)	5306	76.0	80 White	36.7, 30.5–44.5	78.0	ICD codes	15.2	21.0
						20 Other	(median, IQR)				
Young et al (2011) [25]	USA	Prospective cohort	All HIV (+)	5826	79.0	-	40.0, 34.0–46.0	73.0	ICD codes	4.0	8.9
							(median, IQR)				
Hasse et al (2011) [26]	Swiss	Prospetive cohort	All HIV (+)	8444	70.8	-	-	85.0	Self-report	1.5	5.5
Guaraldi et al (2011) [35]	Italy	Case control	All HIV (+)	2854	63.0	-	46.0	-	ICD codes	14.2	-
Yin et al (2010) [27]	USA	Prospective cohort	HIV (+) women	1728	0	13.3 White	40.4, 8.8	65.6	Self-report	8.6	17.9
						56.3 Black	(mean, SD)				
						27.2 Latina					
						3.2 Others					
Triant et al (2008) [28]	USA	Prospective cohort	All HIV (+)	8525	65.2	17.9 Black	-	-	Self-report	2.9	24.9
						55.1 White					
						15.4 Hispanic					
						11.6 Other					

PLWH = people living with HIV; HAART = highly active antiretroviral therapy; ICD = international classification of disease; py = person-years; IQR = interquartile-range; SD = standard deviation

Of the twenty-one studies reporting fracture prevalence, the sample size ranged from 131 in Porcelli et al.’s cross-sectional study in Italy [31] to 96,253 in the retrospective’ study by Byrne et al. in the United States [19] (Table 1). Ethnicity included Caucasian, Black, Hispanic, and Asian subjects. In each study the majority of subjects were men. Subject age, provided by eighteen studies, ranged from 36.7 [24] to 49 [16, 29] years. Sixteen studies provided the percentage of participants that receive highly active antiretroviral therapy (HAART) which ranged from 63% [18] to 100% [19]. Nine studies used self-report [16–18, 22, 26–28, 30, 33], seven studies applied ICD codes [19–21, 23–25, 35] and five studies used X-ray lateral semiquantitative method [6, 29, 31, 32, 34].

Of the thirteen studies reporting fracture incidence, nine were conducted in the United States [17–20, 22, 23, 25, 27, 28], and one each in Spain [21], Denmark [24], and Switzerland [26], with a multinational study reported from Europe [16] (Table 1). The sample size varied from 1,006 in Battalora et al.’s prospective cohort study [17] to 96,253 in a retrospective study by Byrne et al. [19]. In each study, the majority of the subjects were men. Age range and HAART exposure for fracture incidence were similar to studies reporting fracture prevalence. Seven studies used self-report [16–18, 22, 26–28] while six studies used ICD codes to ascertain fracture incidence [19–21, 23–25].

### Bone fracture prevalence

**Fig 2:**
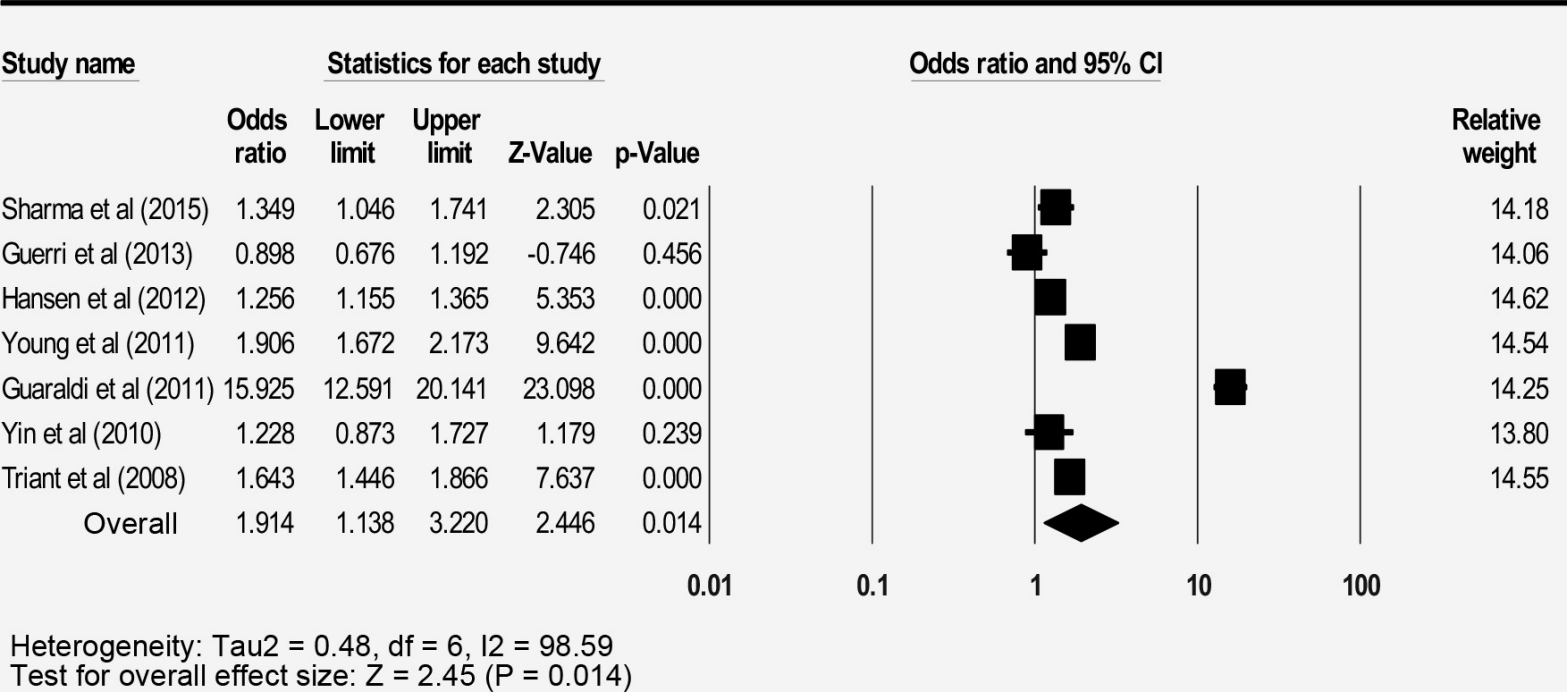
Forest plot for fracture odds ratio between PLWH and general population.

The pooled prevalence of the 21 studies reporting bone fracture among people living with HIV (PLWH) was 6.6% (3.8–11.1) with a high level of heterogeneity (Table 2). The prevalence of PLWH was higher than in the general population with an odds ratio of 1.9 (95% CI: 1.1–3.2) (Fig 2). Males showed a higher prevalence compared to females (6.2% vs 4.9%, respectively). The injecting drugs users (IDUs) PLWH showed the highest prevalence of fracture (8.6%) among the risk group. PLWH who are receiving HAART showed a higher prevalence of fracture compared to those without HAART (6.7% vs 3.5%, respectively).

**Table 2:** Prevalence of fracture among people living with HIV by demographic variable in selected studies.

	Number of studies	Prevalence (%) (95% CI)	Sample size	I^2^ (%)
Overall fracture prevalence	21	6.6 (3.8–11.1)	304309	99.8
**Subgroup analysis**				
Gender				
Female	9	4.9 (3.2–7.4)	9393	91.9
Male	9	6.2 (3.6–10.4)	79632	99.1
Age				
<41 y.o	3	4.4 (1.8–10.4)	4925	97.3
41–50 y.o	3	5.8 (1.9–16.5)	7455	99.2
51–60 y.o	2	7.5 (1.8–26.5)	2614	98.6
>60 y.o	2	7.9 (3.2–18.3)	806	89.8
BMI				
< 18	2	6.4 (5.1–8.2)	990	0.0
18–29.9	3	6.6 (2.5–16.4)	11089	98.3
>29.9	3	4.7 (0.8–24.5)	9579	98.7
HIV risk factor				
Heterosexual	2	6.2 (2.6–13.8)	3822	92.4
IDUs	6	8.6 (4.1–17.0)	22399	97.7
MSM	2	5.5 (2.4–12.0)	5669	96.7
Other risk	2	4.2 (3.0–5.7)	906	0.0
HAART exposure				
With HAART	5	6.7 (3.8–11.3)	20727	98.2
Without HAART	5	3.5 (1.9–6.2)	5210	84.9
Race/ethnicity				
White	5	3.9 (2.6–5.8)	34858	97.4
Black	5	3.3 (2.1–5.0)	5754	86.5
Caucasian	3	3.8 (3.2–4.4)	4531	0.0
Hispanic	4	3.3 (1.6–6.6)	3050	89.7
Others	4	3.0 (2.1–4.3)	3158	0.0
Country region				
European	11	12.0 (7.0–19.7)	20311	98.9
America	9	3.2 (1.6–6.4)	272178	99.8
Multi-countries	1	4.2 (3.8–4.6)	11820	0.0
Fracture ascertainment				
ICD codes	7	3.0 (1.0–8.1)	265215	99.9
Semi-quantitative	5	20.0 (14.6–26.9)	828	79.6
Self-report	9	6.4 (3.6–11.1)	38266	99.2
Study design				
Case control	1	14.2 (13.0–15.5)	2854	0.0
Cross sectional	7	19.2 (15.0–24.1)	1218	75.3
Retrospective cohort	4	1.1 (0.8–0.9)	251229	98.0
Prospective cohort	9	5.4 (3.0–9.5)	49008	99.5

BMI = body mass index; IDUs = inject drugs users; MSM = male sex with male; HAART = highly active anti-retroviral therapy; CI = confidence interval ICD = international classification of disease

### Bone fracture incidence

**Fig 3:**
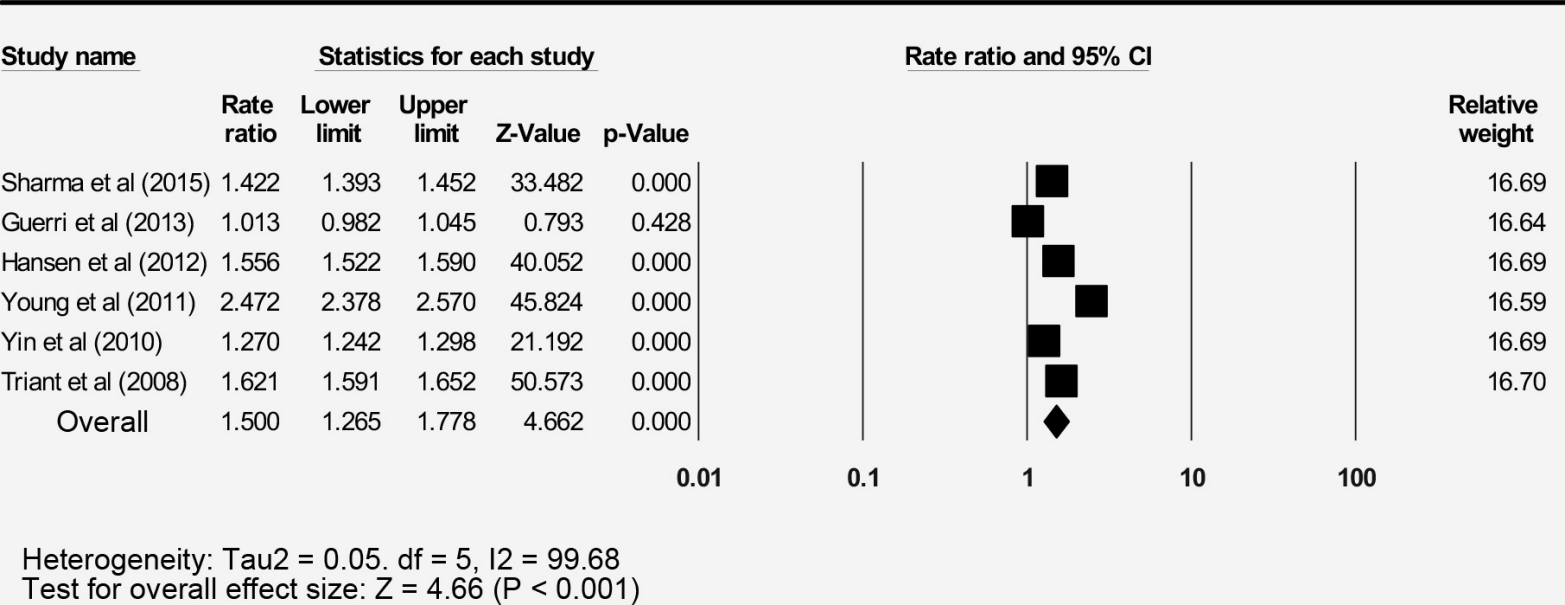
Forest plot for fracture incidence rate ratio between PLWH and general population.

The pooled incidence of the 13 reports bone fracture among people living with HIV (PLWH) was 11.3 per 1000-person years (95% CI: 7.9–14.3) with a high level of heterogeneity (Table 3). The pooled rate ratio was calculated from six studies reporting fractures of the control group as 1.5 (95% CI: 1.3–1.8) (Fig 3).

**Table 3:** Incidence of fracture among people living with HIV by demographic variable in selected studies.

	Number of studies	Incidence (per 1000 py) (95% CI)	Sample size	I^2^ (%)
Overall fracture incidence	13	11.3 (7.9–14.5)	300237	99.9
*Subgroup analysis*				
Country region				
European	3	11.5 (4.1–18.9)	16239	99.9
America	9	12.0 (7.8–16.3)	272178	99.9
Multi-countries	1	7.2 (7.0–7.4)	11820	0.0
Fracture ascertainment				
ICD codes	6	7.3 (4.0–10.7)	262361	99.9
Self-report	7	15.2 (6.2–12.1)	37876	99.9
Study design				
Retrospective cohort	4	3.5 (1.7–5.4)	251229	99.9
Prospective cohort	9	15.1 (10.0–20.2)	49008	99.9
Overall fracture incidence	13	11.3 (7.9–14.5)	300237	99.9
*Subgroup analysis*				
Country region				
European	3	11.5 (4.1–18.9)	16239	99.9
America	9	12.0 (7.8–16.3)	272178	99.9
Multi-countries	1	7.2 (7.0–7.4)	11820	0.0
Fracture ascertainment				
ICD codes	6	7.3 (4.0–10.7)	262361	99.9
Self-report	7	15.2 (6.2–12.1)	37876	99.9
Study design				
Retrospective cohort	4	3.5 (1.7–5.4)	251229	99.9
Prospective cohort	9	15.1 (10.0–20.2)	49008	99.9

ICD = international classification of disease; py = person-years; CI = confidence interval

### Fracture risk factors

Traditional risk factors for fracture identified among PLWH included older age (> 60 years), smoking, and osteoporosis with adjusted hazard ratios (aHR) of 1.4, 1.3, 3.3, respectively (Table 4). HCV co-infection was an independent HIV-related risk factor with an adjusted hazard ratio of 1.6 (95% CI: 1.3–1.9).

**Table 4:** Risk factor of fractures among people living with HIV.

Risk factor	Sample size	Adjusted Hazard Ratio (CI 95%)	I^2^	P value
Older age (>60 years)	32302	1.4 (1.3–1.6)	57.73	0.001**
Female gender	12038	1.2 (0.9–1.5)	0.00	0.258
Nadir CD4 cells count	23858	1.0 (0.9–1.1)	7.89	0.844
Smoking	18032	1.3 (1.1–1.5)	66.41	0.010*
HCV coinfection	18652	1.6 (1.3–1.9)	0.00	0.001**
Lower BMI	12826	1.2 (0.9–1.8)	85.19	0.281
Prior fracture	12826	2.2 (0.7–7.1)	87.58	0.168
Osteoporosis	12826	3.3 (2.2–5.1)	0.00	0.001**

CI = confidence interval; HCV = Hepatitis C Co-infection; BMI = body mass index

* refers to p <0.05

** refers to p < 0.005

### Meta-regression analysis and publication bias

**Fig 4:**
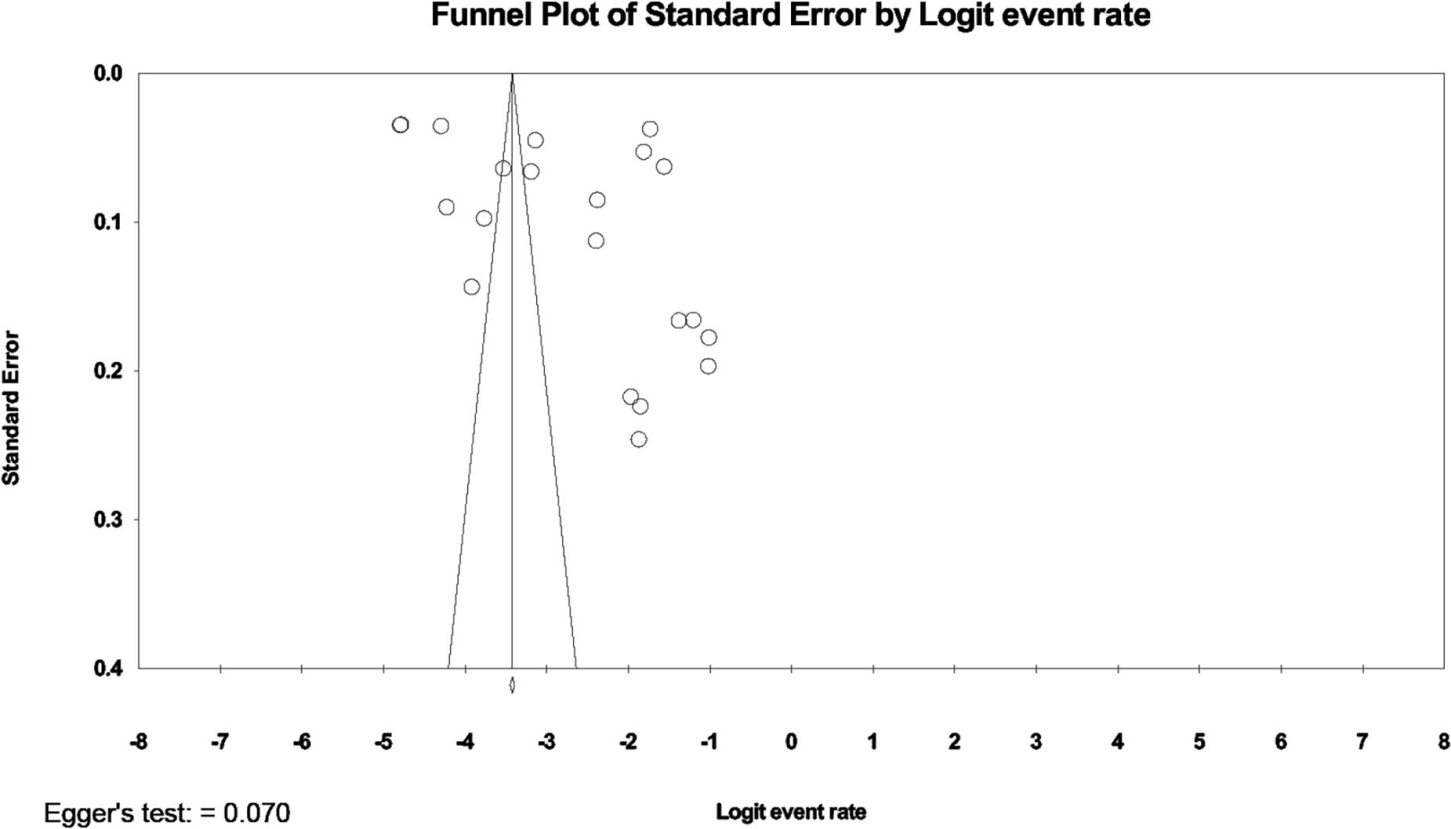
Funnel plot for publication bias test across studies.

A high level of heterogeneity existed across the studies reporting prevalence (I^2^ = 99.8%, Table 2). Univariate analysis was conducted to find significant independent covariates as a potential source of heterogeneity that could be included in a meta-regression model (Table 5). The covariates of study design (p < 0.001) and fracture identification method (p < 0.05) showed significant coefficient regression. After these covariates were included in the meta-regression analysis, only study design covariates provided a significant coefficient regression (p < 0.05). Publication bias was analyzed by generating a funnel plot and Egger’s test with neither demonstrating evidence of asymmetry (Fig 4).

**Table 5:** Meta-regression analysis of fracture risk factors affecting heterogeneity on prevalence ^a^.

Variable	Univariate coefficient (95% CI)	P value	Multivariate Coefficient (95% CI) ^b^	P value
Country region				
Reference: America				
Europe	1.4 (0.5–2.3)	0.003*	0.1 (-0.9–0.9)	0.994
Multi-country	0.3 (-1.9–2.5)	0.807	-0.2 (-1.7–1.4)	0.836
Study design				
Reference: Retrospective cohort				
Case control	2.6 (1.1–4.2)	P < 0.001*	2.6 (0.9–4.4)	0.003*
Cross-sectional	3.0 (2.1–3.9)	P < 0.001*	3.4 (1.3–5.4)	0.001*
Prospective cohort	1.6 (0.7–2.4)	P < 0.001*	2.0 (0.7–3.3)	0.002*
Fracture ascertainment				
Reference: ICD codes				
Self-report	0.8 (-0.5–2.1)	0.217	-0.5 (-1.7–0.7)	0.402
Semi-quantitative	2.1 (0.6–3.6)	0.006*	-0.3 (-2.0–1.4)	0.722

* refers to statistically significant at p < 0.05

^a^ refers to I^2^ statistic result (99%)

^b^ refers to Confidence Interval

## Discussion

The findings of this study revealed that people living with HIV have an increased risk of bone fracture, 1.9 times higher than that observed in the general population. This finding was higher than a systematic review of HIV postmenopausal women that reported a fracture prevalence 1.5 times greater than HIV negative postmenopausal women [36]. The greater percentage of males in the studies included in the current study may explain the difference.

People living with HIV experienced an incidence rate of fracture 1.5 times that of the general population. Dong and colleagues’ meta-analysis reported a similar incidence rate ratio of fracture among PLWH of 1.8 [11]. However, the analysis only focused on subjects with HIV and HCV co-infection which may have yielded a higher incidence rate ratio. Maalouf et al. have reported a higher risk of fracture among patients with HIV/HCV co-infection compared to HIV mono-infection which was identified as a risk factor in the current study [20].

The four risk factors found significant in this meta-analysis have previously reported including older age, osteoporosis, smoking and HCV co-infection. A meta-analysis by Shiau et al. identified similar results but did not report pooled hazard ratios [37]. Older age as a traditional risk factor in this study was reported in a meta-analysis by Dong et al. who studied risks among HIV and HCV co-infected patients [11]. Osteoporosis as a risk factor for fractures showed a lower pooled adjusted hazard ratio than Short et al. [38] who reported that HIV infected men with osteoporosis had a 3.5-fold risk of fracture compared to those without osteoporosis. However, the limited population of the study may have overestimated the findings. Smoking, a significant and modifiable fracture risk factor among PLWH, is associated with a bone mineral density which is influenced by both dose and duration of smoking [39]. Wu et al. [40] reported that smokers had twice the risk of fracture compared to non-smokers. Smoking cessation programs among smokers with HIV may reduce fracture risk after just three months [41]. The HCV co-infection risk, with pooled aHR of 1.6, confirms the findings of Dong et al’s meta-analytic study. [11]. Another meta-analytic study by Shiau et al. [37] also found that HCV co-infection was an independent risk factor for both fragility and non-fragility fractures. Early intervention for HCV co-infection and PLWH, recommended by Bedimo et al. is critical to reducing bone turnover [42]

The current study adds to the literature by reporting both the prevalence and incidence rate of fractures including the HIV-related risk factor for fracture among people living with HIV. The studies included in the meta-analysis included sample sizes exceeding 1,000 and a diverse population of patients with HIV. Previous reviews focused on smaller samples with at-risk populations. Finally, a pooled adjusted Hazard Ratio of fracture risk factors provide more specific analysis than a simple summary of factors.

### Limitations

The literature search excluded non-English reports as the studies all originated in developed countries, fracture risk factors of less developed countries may differ. Despite subgroup and meta-regression analysis, the synthesized literature revealed significant heterogeneity.

## Conclusions

A comprehensive meta-analysis revealed a higher prevalence and incidence rate of fracture among PLWH compared to the general population. Smoking, older age, osteoporosis, and HCV coinfection are significant risk factors for fracture among PLWH.

Our finding highlights a higher risk of fracture among PLWH compared to the general population. Osteoporosis, smoking and HIV/HCV coinfection as the significant modifiable risk factors should be prioritized by the HIV health provider when managing PLWH. Further investigation of the interactions among risk factors is urgently needed to prevent bone fractures.

## Supporting information

S1 Checklist PRISMA 2009 checklist. (DOC)

S1 Table Quality appraisal using the JBI checklist tool (n = 21). (DOCX)
