# Supplementary material for: Bone fracture among people living with HIV: A systematic review and meta-regression of prevalence, incidence, and risk factors
Source: PLoS One. 2020 Jun 4;15(6):e0233501. doi: 10.1371/journal.pone.0233501 (PMC7271989; doi:10.1371/journal.pone.0233501)
Supplement: S1 Table — (DOCX) [file pone.0233501.s002.docx]

Table 1. Quality appraisal using the JBI Checklist Tool (n=21)

| Author (year) | Sample Frame | Sample recruitment | Sample size | Subject & Setting | Sufficient coverage of the sample | Valid method | Reliable measure | Appropriate statistical analysis | Response Rate | Total met criteria |
| --- | --- | --- | --- | --- | --- | --- | --- | --- | --- | --- |
| Ciulini et al (2017) | Y | Y | Y | Y | Y | Y | Y | Y | Y | 9 |
| Borges et al (2017) | Y | Y | Y | Y | Y | Y | Y | Y | N/A | 8 |
| Battalora et al (2016) | Y | Y | Y | Y | Y | Y | Y | Y | N/A | 8 |
| Sharma et al (2015) | Y | Y | Y | Y | Y | Y | Y | Y | U | 8 |
| Gazzola et al (2015) | Y | Y | Y | Y | Y | Y | Y | Y | U | 8 |
| Byrne et al (2015) | Y | Y | Y | Y | Y | Y | Y | Y | N/A | 8 |
| Short et al (2014) | Y | Y | Y | Y | Y | Y | Y | Y | U | 8 |
| Porcelli et al (2014) | Y | Y | Y | Y | Y | Y | Y | Y | U | 8 |
| Borderi et al (2013) | Y | Y | Y | Y | Y | Y | Y | Y | N/A | 8 |
| Peters et al (2013) | Y | Y | Y | Y | Y | Y | Y | Y | U | 8 |
| Maalouf et al (2013) | Y | Y | Y | Y | Y | Y | Y | Y | N/A | 8 |
| Guerri et al (2013) | Y | Y | Y | Y | Y | Y | Y | Y | Y | 9 |
| Yin (2012) | Y | Y | Y | Y | Y | Y | Y | Y | U | 8 |
| Torti (2012) | Y | Y | Y | Y | Y | Y | Y | Y | U | 8 |
| Lo Re et al (2012) | Y | Y | Y | Y | Y | Y | Y | Y | U | 8 |
| Hansen et al (2012) | Y | Y | Y | Y | Y | Y | Y | Y | Y | 9 |
| Young et al (2011) | Y | Y | Y | Y | Y | Y | Y | Y | N/A | 8 |
| Hasse et al (2011) | Y | Y | Y | Y | Y | Y | Y | Y | Y | 9 |
| Guaraldi et al (2011) | Y | Y | Y | Y | Y | Y | Y | Y | N/A | 8 |
| Yin (2010) | Y | Y | Y | Y | Y | Y | Y | Y | U | 8 |
| Triant et al (2008) | Y | Y | Y | Y | Y | Y | Y | Y | N/A | 8 |

Y= yes; U=unclear; N/A=not applicable
